# Decolonising audiology education: Epistemic barriers and opportunities for Black African students

**DOI:** 10.4102/sajcd.v73i1.1152

**Published:** 2026-04-08

**Authors:** Musa Makhoba, Sarasvathie Reddy, Mershen Pillay

**Affiliations:** 1 Discipline of Audiology, School of Health Sciences, University of KwaZulu-Natal, Durban, South Africa; 2 Discipline of Education and Development Studies, College of Humanities, University of KwaZulu-Natal, Durban, South Africa; 3 Massey University, Auckland, New Zealand; 4 Discipline of Speech and Language Pathology, College of Health Sciences, University of KwaZulu-Natal, Durban, South Africa

**Keywords:** BAFLS, teaching, assessment, learning, experienced curriculum, recontextualisation

## Abstract

**Background:**

In South Africa, the Audiology curriculum remains rooted in Eurocentric epistemologies that do not reflect the lived realities, languages and cultural knowledge systems of Black African First-Language Speaking (BAFLS) students. This study responds to national calls for epistemic transformation in health sciences education, including Audiology.

**Objectives:**

This study examined the epistemological challenges encountered by BAFLS Audiology students at the University of Interest (UoI). Our objectives were to explore how the BAFLS students experience accessing and navigating the undergraduate Audiology curriculum and recommend interventions to address the identified challenges.

**Method:**

A qualitative hermeneutic phenomenological design informed the methods adopted. Ten purposively selected BAFLS graduates from the UoI participated in in-depth semi-structured interviews. Cowen’s Logical Model of Curriculum Development (LMCD) framed the data generation and analysis. A decolonial perspective emerged naturally in the interpretation of the findings, based on the experiences reported, which the authors incorporated into the discussion of the recommendations for improvements.

**Results:**

Thematic analysis revealed a lack of alignment between some of the curriculum content taught and the realities of Audiology practice in African contexts. Assessment practices were experienced as biased and failing to account for the students’ linguistic and cultural diversity. Experienced linguicism, racism and classism contributed to surface learning and graduates feeling underprepared to provide contextually relevant and Afrocentric professional care.

**Conclusion:**

The undergraduate Audiology curriculum at the UoI needs a fundamentally transformative redesign towards being more Afrocentric and epistemologically inclusive towards BAFLS students.

**Contribution:**

This study provides a pathway towards the decolonisation of the Audiology curriculum by identifying specific challenges to be addressed urgently to improve its epistemic inclusivity towards BAFLS students, who currently feel marginalised.

## Introduction

Despite longstanding calls for greater racial, linguistic and epistemological inclusivity in Audiology training, clinical practice and research, these demands remain largely unmet in South Africa (Abrahams et al., 2019; Pillay et al., 1997). While some progress has been made in transforming the profession to reflect the country’s demographics, the Audiology curriculum continues to be shaped by Eurocentric epistemologies dating back to its establishment in 1938 (Beecham, 2002; Khoza-Shangase & Mophosho, 2018; Uys & Hugo, 1997). This entrenched epistemic orientation often overlooks the lived experiences, cultural identities and knowledge systems of Black African First-Language (isiZulu) Speaking (BAFLS) students.

The students’ experiences of the curriculum in Speech-Language and Audiology discipline generally need attention in research. However, this research gap appears notably more pronounced when considering the diverse racial and ethnic groups of students. Addressing the research gap from a specific racial perspective not only contributes to a better understanding of the phenomenon of epistemic transformation or the impact of its absence but also allows for a unique understanding of BAFLS students’ experiences, a group that appears to be more vulnerable, as indicated by the current study’s findings. It is paramount that the experiences of the group that seems to be at the receiving end of an epistemic exclusion (BAFLS) within Audiology education are explored in order to advance their voices that may have been previously silenced for far too long (Abrahams et al., 2023), in particular, the experienced curriculum, how students encounter teaching and learning and the hidden curriculum. Further, implicit norms and values promoted through the undergraduate curriculum in South Africa have also received minimal attention (Bitzer & Botha, 2011; Hafferty et al., 2015).

This study explores how BAFLS Audiology graduates experienced their professional development within an undergraduate curriculum that arguably remains untransformed in its epistemological foundations (Moonsamy et al., 2017). In this article, we highlight the epistemological disjunctures that left these students feeling invisible and marginalised and propose strategies for radical decolonisation aimed at fostering meaningful, student-informed curriculum transformation. For the transformation to be radical, it must be practically implementable, with the intention of creating an impactful, time-bound and measurable shift that disrupts the status quo.

The findings address the identified challenges related to the lack of epistemic transformation in contextually similar institutions. The study is designed to inform future curriculum reviews that promote Afrocentric and epistemologically inclusive approaches at the University of Interest (UoI) (Pseudonym) and similar institutions. Informed by the interpretations of the participants’ lived experiences, this work offers critical insights for professional councils, associations and other relevant stakeholders, urging them to address the ways in which current curricula perpetuate marginalisation and produce graduates who feel ill-equipped for practice in culturally and linguistically diverse contexts.

### The audiology curriculum within the South African Higher Education context

The *experienced undergraduate curriculum* is understood here as the co-constructed learning environment shaped by students, lecturers and other stakeholders, including Department of Health audiologists, the quality assurance entities, the professional council and professional associations, through teaching, learning and assessment processes aimed at achieving specified educational outcomes (Reddy, 2018, p. 163). These stakeholders influence what forms of knowledge are legitimised and privileged within the undergraduate Audiology curriculum. However, current efforts by these actors appear insufficient in producing a meaningful epistemic shift that fosters fundamental inclusion for Black African First-Language Speaking (BAFLS) students, as evidenced by the findings of this study.

Audiology, introduced in South Africa in 1938 as a Euro-American model of rehabilitation, has expanded significantly from its origins in two historically White universities. Today, five universities offer undergraduate programmes in Audiology, admitting students from more racially, linguistically and culturally diverse backgrounds (Pillay & Kathard, 2015; Swanepoel, 2006). Table 1 outlines the institutions and the programmes offered. This expansion indicates progress in demographic access but does not necessarily reflect epistemological transformation of the curriculum (Moonsamy et al., 2017; Pillay et al., 2020).

**TABLE 1:** Universities offering undergraduate Audiology programmes in South Africa.

Historical Institutional Identity	University	Programme offered	Province
Former White Afrikaans (FWA)	University of Pretoria	BA: Audiology	Gauteng
Former White English (FWE)	University of the Witwatersrand	BA: Speech and Hearing Therapy	Gauteng
Former White English (FWE)	University of Cape Town	BSc: Audiology	Western Cape
Former Black African (FBA)	Sefako Makgatho Health Sciences University	BSc: Speech Language Pathology and Audiology	Limpopo
Former Black (Indian) and FWE	University of KwaZulu-Natal	Bachelor of Audiology	KwaZulu-Natal

*Source*: Adapted from Swanepoel, D.W. (2006). Audiology in South Africa. *International Journal of Audiology, 45*(5), 262–266. https://doi.org/10.1080/14992020500485650; Bunting, I. (2006). The higher education landscape under apartheid. In N. Cloete, P. Maassen, R. Fehnel, T. Moja, T. Gibbon, & H. Perold (Eds.), *Transformation in Higher Education: Global Pressures and Local Realities* (pp. 35–52). Springer Netherlands. https://doi.org/10.1007/1-4020-4006-7_3

BA, Bachelor of Arts; BSc, Bachelor of Science.

Despite increased access, the core curriculum in Audiology remains epistemologically Eurocentric, with limited integration of African knowledge systems and culturally responsive pedagogies (Abrahams et al., 2019; Khoza-Shangase & Mophosho, 2018). Eurocentric clinical and research frameworks continue to dominate, often privileging reductionist, positivist methodologies that marginalise indigenous approaches to hearing, communication and wellness (Penn, 2007; Pillay & Kathard, 2015). Consequently, the curriculum fails to adequately prepare graduates to serve the diverse linguistic and cultural needs of the South African population.

This epistemic exclusion is not unique to Audiology. The broader South African higher education landscape remains largely untransformed in its curriculum, over 30 years into democracy (Heleta, 2016; Mbembe, 2016). Many curricula have and continue to retain their Western colonial structures, often reproducing a hegemonic knowledge hierarchy that privileges whiteness and Eurocentrism, while marginalising Black students and African epistemologies (Heleta & Dilraj, 2024; Jawitz, 2012; Pinar, 2010; Ramoupi, 2014).

In such contexts, BAFLS students are forced to adapt to dominant paradigms that are incongruent with their lived realities and linguistic repertoires. This often results in alienation, surface learning and feelings of inadequacy, as revealed in this study and others (Pillay & Serooe, 2019; Soudien, 2010). As scholars increasingly call for a fundamental restructuring of curricula to disrupt these colonial logics (Menon & Castrillón, 2019), there is an urgent need to rethink how Audiology and other health professions are taught. In response to this call, the current study investigates two core questions:

- *How do BAFLS students experience accessing and navigating the Audiology curriculum?*
- *What transformative interventions are necessary to address the challenges identified?*

### The study framework

**FIGURE 1:**
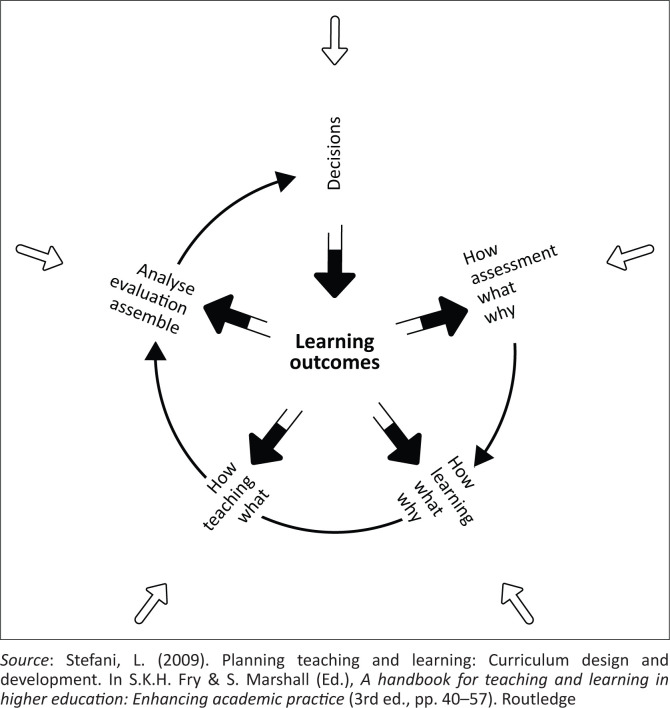
Stefani’s modification of J. Cowen’s Logical Model of Curriculum Development.

From inception, the current study was located within an interpretive paradigm, within which the data were generated. However, the emergent findings suggested a need for a more decolonial stance in their interpretation and discussion, as seen in the discussion section of this paper. Within this context, this study was conceptually framed by the Logical Model of Curriculum Development (LMCD), initially conceptualised by Cowan and Harding (1986) and later modified by Stefani (2009). The LMCD provides a comprehensive framework for examining the dynamic interactions between curriculum components, namely intended learning outcomes, teaching, learning and assessment, within broader institutional and professional contexts (Figure 1). Hermeneutic phenomenology provided a lens to interpret how such dynamics are experienced by the students.

The LMCD posits that curriculum development and student learning are shaped by four interrelated forces:

1. **External influences:** The curriculum is subject to external drivers such as national health policies, professional councils (e.g. the Health Professions Council of South Africa [HPCSA]) and clinical practice settings. These forces significantly impact how teaching, learning and assessment are framed within the Audiology programme (Cowan & Harding, 1986; Du Toit, 2011; Stefani, 2009).
2. **Learning outcomes:** The intended outcomes, defined in terms of knowledge, skills and attitudes, form the foundation upon which teaching and assessment strategies are constructed. These outcomes also shape how students perceive and engage with their learning experiences (Harris & Clayton, 2019).
3. **Assessment:** Assessment practices (e.g. tests, exams, clinical evaluations) exert a powerful influence on students’ approaches to learning. The literature confirms that the nature of assessment can either promote deep learning or encourage surface learning strategies (Heeneman et al., 2015).
4. **Teaching and learning processes:** While teaching can create opportunities for learning, it does not guarantee that learning occurs. The LMCD recognises that learning is a constructivist and student-centred process that must be aligned with both assessment methods and curricular goals.

In this study, these four dimensions informed the design of the data production instruments and the thematic analysis process. Interview questions were mapped against each component of the LMCD to explore how BAFLS graduates experienced the curriculum in relation to teaching, learning, assessment and external professional demands. The framework, applied within hermeneutic phenomenology, thus enabled a systematic interrogation of the curriculum’s alignment, or lack thereof, with the needs and epistemic positioning of BAFLS students.

## Research methods and design

This qualitative study was situated within an interpretive paradigm, which prioritises understanding participants’ lived experiences and the meanings they attach to phenomena. We employed a hermeneutic phenomenological design to explore the epistemological experiences of BAFLS Audiology graduates. Using purposive sampling, 10 graduates from the UoI were selected based on their completion of the undergraduate Audiology programme and having worked professionally in the same province for between 3 years and 5 years post-graduation, ensuring their reflections on the curriculum and clinical practice were both recent and informed (see Table 2 for participant demographics). Data were collected through in-depth, semi-structured interviews guided by an interview schedule developed according to the study’s theoretical framework, the LMCD (Figure 1).

**TABLE 2:** Participant demographics.

Participant	Gender	First language	High school background	Language of communication at school	Racial schooling background
Participant 1	Male	isiZulu	Rural	Zulu medium School	Black African
Participant 2	Female	xiTsonga - English (isiZulu 2nd language)	Urban	English medium school	Multiracial
Participant 3	Female	Zulu	Urban	English medium school	Multiracial
Participant 4	Female	Nyanja-English (IsiZulu-2nd language)	Urban	English medium school	Multiracial
Participant 5	Female	IsiZulu	Rural	Zulu medium Schooling	Black African
Participant 6	Female	IsiZulu	Urban	Zulu medium	Black African
Participant 7	Female	IsiZulu	Urban	Zulu medium	Multiracial
Participant 8	Female	IsiZulu	Rural	Zulu medium	Black African
Participant 9	Female	IsiZulu	Urban	English medium	Multiracial
Participant 10	Female	IsiZulu	Urban	English Multiracial Schooling	Multiracial

### Data analysis

All interviews were audio-recorded and transcribed verbatim to preserve participants’ original expressions (Crowther et al., 2017). A qualified bilingual language expert (PhD level), fluent in isiZulu and English, translated all isiZulu portions into English to maintain linguistic and contextual accuracy (Garraio et al., 2022). The transcripts were imported into NVivo 12 software for systematic management and analysis. Initially, deductive content analysis was used to organise data into broad parent themes derived from the theoretical framework (Erlingsson & Brysiewicz, 2017). Subsequently, thematic analysis was conducted within these categories to inductively identify emergent sub-themes, following the six-step process outlined by Braun and Clarke (2006) and adapted by Erlingsson and Brysiewicz (2017).

### Trustworthiness

To ensure the study’s trustworthiness, rigorous procedures were followed throughout data generation and analysis (Armour et al., 2009). The principal researcher’s prior personal and professional engagement with the phenomenon provided deep contextual understanding and reflexive insight, enhancing credibility (Crowther et al., 2017). The principal researcher, who conducted the study, is a first-language isiZulu speaker and trains students, having prior experience with a curriculum similar to that of the current study participants, as an undergraduate student. The two other authors are Indian, first-language English speakers, and have trained students at both undergraduate and postgraduate levels, some of whom experienced similar struggles to those shared by the participants. All the researchers embraced their deep awareness of some of the students’ struggles and their own deep insider-outsider perspectives, in line with hermeneutic phenomenology, which does not encourage objectivity in the analysis and interpretation of the data (Godden & Kutsyuruba, 2023; Ramsook, 2018). Reflexivity was further maintained through reflective journaling and transparent documentation of analytic decisions, supporting confirmability and dependability (Dangal & Joshi, 2020). All the researchers have previously supervised and conducted interpretive research and are deeply knowledgeable in the nuances of decolonial research. This was an advantage in terms of preparing, conducting the study and interpreting the findings, while avoiding a paradigmatic conflict, as this interpretive study evolved into a more decolonial one post data analysis.

While member checking is known as a quality measure in qualitative research, it diverges from the principles of Heideggerian hermeneutic phenomenology and is therefore not advisable for this design. For instance, it is understood that the participant’s own interpretation of their lived experiences can be evolved over time. Seeking a fixed recount of their lived experiences conflicts with hermeneutic phenomenology or offers minimal, if any value (Harvey, 2015; McConnell-Henry et al., 2011; Vella, 2024). For these reasons, the current study omitted member-checking, as did Alsaigh and Coyne (2021). Instead, peer debriefing and audit trails were employed to verify the analysis (Ahmed, 2024). These strategies collectively aimed to produce a rich, trustworthy representation of participants’ experiences.

### Ethical considerations

Ethical clearance was granted by the UOI’s Humanities and Social Sciences Ethics Committee (reference number: HSS/2026/018m), the Provincial Health Research Ethics Committee (reference number: KZ_201812_003) and gatekeeper permission was obtained from the Registrar’s office prior to data generation. Participants provided written informed consent after receiving full information about the study’s purpose, confidentiality and voluntary participation. All data were anonymised and stored securely in password-protected files to ensure participant confidentiality and data integrity throughout the research process.

## Results

This study explored how BAFLS graduates from the UOI experienced accessing the undergraduate Audiology curriculum through teaching, learning and assessment.

Participants (*N* = 10) were all qualified Audiologists working in public hospitals in the province where the UoI is located, with 3–5 years of post-graduation clinical experience. The participants included predominantly isiZulu first-language speakers, with one xiTsonga and one Nyanja speaker, both of whom were also fluent in isiZulu. Six had attended multiracial high schools, while four came from predominantly Black African schooling backgrounds, reflecting diverse socio-linguistic and educational contexts.

Emergent themes are reported within three overarching themes aligned to the LMCD: (1) Accessing Audiology through teaching, (2) Accessing Audiology through assessment and (3) Accessing Audiology through learning. Each is discussed below.

### Accessing audiology through teaching

Participants described a misalignment between what was taught and the realities of clinical practice. Three interrelated subthemes emerged: poor knowledge recontextualisation, curricular parochialism and omission of Afrocentric content.

#### Poor recontextualisation of professional knowledge

Participants perceived the curriculum as overly idealistic and detached from South African clinical realities. Content was experienced as rigid and formulaic, hindering graduates’ ability to manage complex patient scenarios. As one participant noted:

‘In university, it’s clear-cut, like when they [*lecturers*] give you a question, they already know what they are looking for. But when you get a patient [*in the field of practice*], sometimes you just get a case where you don’t know where the problem is. It’s much more diverse [*in practice*] than what you are learning in your books. They [*lecturers*] make it very structured and very limited’. (Participant 4, Female, isiZulu first language speaker)

This disconnect resulted in feelings of under-preparedness, echoing concerns in existing literature about the failure to adapt professional knowledge into relevant curriculum content (Khoza-Shangase & Mophosho, 2018).

#### Parochial and contextually irrelevant content

The curriculum was perceived as narrowly focused on Eurocentric ideals and global Northern institutions, often to the exclusion of South African realities:

‘What I learnt in varsity and the reality are two different things. I mean, we learnt about Gallaudet University in sign language. I mean, where is Gallaudet University right now? We don’t even have sign language schools in South Africa. The context wasn’t linking … And it [*the curriculum content*] was more of the ideal developed country than for our own context.’ (Participant 2, female, xiTsonga first language speaking)

Such content was seen as aspirational but contextually irrelevant, failing to prepare students for resource-constrained environments they met in clinical practice.

#### Omission of Afrocentric worldviews

Participants noted that the curriculum marginalised indigenous cultural practices, spiritual beliefs and healing systems:

‘… And even exploring therapy outcomes. It was more rigid in terms of “this is the way and there was no other way of… as a Black person, how do you deal with it [*providing therapy*]”. Are there other alternative ways that a person should explore first or consider? So, I don’t think it [*the teaching*] prepared us much for the Black community that we were going to serve.’ (Participant 2, female, xiTsonga first language speaking)

The omissions in the above excerpt are reflective of the ‘null curriculum’ (Le Grange, 2016), which suggests epistemic exclusion of alternative approaches to providing Audiology services. This is essentially an exclusion of Afrocentric ways of care while prioritising Eurocentric dominance within the curriculum.

### Accessing audiology through the assessment

Assessment practices were widely perceived as biased and non-transparent. Two themes emerged: epistemic injustice and credibility deficits, as well as power imbalances during clinical evaluations.

#### Epistemic injustice and biased grading

Several participants reported being graded in a manner that does not truly reflect their academic improvements. They report that they consistently receive a particular range of marks regardless of efforts towards improvement, attributing this to racial or linguistic prejudice:

‘You end up getting the same mark, but I’m working extra hard, how am I still getting the 60? That’s the other thing, you just don’t know how you’re not improving. You feel like you’re working harder, but you’re still getting the exact same mark.’ (Participant 4, female, isiZulu first language speaker)

Participants perceived that their language background and race influenced grading more than the quality of their work, a phenomenon associated with epistemic injustice (Fricker, 2007).

A participant elaborated on her experiences of epistemic injustices when she used isiZulu in her clinical session with a patient:

‘Remember, we used to have observation rooms. And I would observe a colleague’s session, and I feel like I did better, but the Indian or White counterpart will get a better mark. Then I think, how did you [*the clinical supervisor*] critique me because, for the whole session, you did not understand it?.’ (Participant 9, female, isiZulu first language speaking)

#### Linguicism and class-based disadvantage

Students from working-class and non-English-speaking backgrounds faced additional challenges. Language barriers were linked to misinterpretation of instructions and being judged as less competent in their clinical performance:

‘I felt like other people [*First Language English Speakers*] had an advantage because of the language and race. I think we [*BAFLS students*] were put in a disadvantaged position. When you want to converse with clients, there are things that you want to say, but because of the language, you would sometimes realise that you can’t fully express yourself and explain some of the things to the patients.’ (Participant 5, Female, isiZulu first language speaking)

This aligns with findings from Mahboob and Szenes (2010) that linguicism intersects with socioeconomic disadvantage in higher education. Participants also reported financial inequities influencing performance:

‘Then you would see other students coming to clinics with the Marries [*biscuits*] and stuff like that, and smarties [*sweets*]. They would get more marks. And then you’re like, must I have to choose between eating my food later and giving it to this child [*patient*] now because I want marks.’ (Participant 8, Female, IsiZulu first language speaking)

Such examples underscore the covert classism embedded in assessment practices. Those who could afford to buy extra materials for the clinic stood a chance of being rewarded with better marks than those who could not.

#### Abuse of power during clinical supervision

Clinical supervisors were reported to use intimidating tactics that impaired learning. Feedback during sessions was sometimes public and shaming:

‘It was more of “Do you know what you’re doing? What did you just do that?” So, more of being intimidated throughout the whole session because you are Black. So, it was tough.’ (Participant 2, female, xiTsonga first language speaking)

This emotional distress led to decreased clinical performance, reinforcing the need for sensitive and supportive supervision (McNamara et al., 2017).

### Accessing audiology through learning

Participants described two dominant learning experiences: surface learning and contextually misaligned curriculum content.

#### Surface learning and lack of integration

Several participants reported learning ‘just to pass’, with limited understanding or integration of knowledge:

‘It’s just like I am studying to pass. Until I have to apply it. Like I remember, [*module name*]. I used to pass it very well, but I didn’t understand it. All those waves and how they move.’ (Participant 5, female, isiZulu first language speaking)

Others echoed this sentiment, citing pressure and overwhelming content loads as reasons for surface-level engagement.

#### Contextual misalignment and cultural disconnection

Participants felt their learning experiences ignored the lived experiences and cultural values of Black South African communities:

‘So, what we learnt, wasn’t for the Black African first language. The context was more of the ideal, developed country. I think everything was under a “White context”. Because I mean with Black people, first of all there was no black child or any class or any module that relates to Black culture, Black history or counselling a Black person. With our learning, it always has to … eye contact matters, with Black people, eye contact doesn’t matter. Actually, it’s even more disrespectful for one to look for eye contact. Always in our profession, we fail you if you don’t have eye contact. We automatically think there’s something wrong if you don’t have eye contact.’ (Participant 2, female, xiTsonga first language speaking)

This disconnection undermined the students’ preparedness to work in multilingual, multicultural clinical settings, raising critical concerns for curriculum transformation and cultural responsiveness.

## Discussion

This study examined the experiences of BAFLS as they navigated the undergraduate Audiology curriculum at a South African university. The study was conducted as conceptually framed by the Logical Model of Curriculum Design (Figure 1), within the interpretive hermeneutic phenomenology design. The emergent findings suggested a need for a decolonial turn in how the authors interpreted them, as they could not be justifiably and sufficiently interpreted outside of a decolonial theory, based on the reported experiences. In this section, we therefore move beyond hermeneutic interpretation and adopt a decolonial interpretive stance, guided by a decolonial theory as an interpretive frame, which seemed the best fitting for several reasons. Decolonial research (or theory) concerns itself with exposing the damage of colonisation, along with its persistent negative legacies (Naicker, 2023; Parveen, 2025). Furthermore, decolonial research should reflect the experiences of victims of colonisation or neocolonisation in its proposals for meaningful or fundamental freedom from persistent colonial legacies or neo-coloniality (Ndlovu-Gatsheni, 2015). The current study findings align with these criteria, underscoring the need to adopt a decolonial approach in this discussion section. Furthermore, decolonial theory should be adopted in the critique of post-colonial efforts and their lack of material impact, where the effects are seen as minimal (Fosu, 2025). This further prompted the authors to engage with the decolonial critique of the findings, drawing on the work of several decolonial scholars.

On the surface, the findings reveal significant challenges in teaching, assessment and learning that undermine epistemological access and the professional preparation of BAFLS students. Upon deeper interpretation, the findings suggest that the undergraduate Audiology curriculum was experienced as Eurocentric, linguistically exclusive and misaligned with the clinical realities and cultural needs of South Africa’s diverse populations. These challenges contributed to surface learning and perceived under-preparedness for practice, particularly in underserved, multilingual and culturally diverse communities.

### Epistemic misalignment and curricular eurocentrism

Participants consistently reported that the Audiology curriculum did not reflect the complexities of clinical practice in the South African context. Instead, it prioritised knowledge systems and examples derived from Global North contexts, failing to incorporate Afrocentric health beliefs or local public sector conditions. This finding echoes broader critiques of the South African higher education system’s persistent Eurocentrism (Heleta, 2016; Heleta & Dilraj, 2024; Ndlovu-Gatsheni, 2013). As such, the undergraduate Audiology curriculum content and structure, as currently designed, served a role of an epistemicide, an epistemically violent replacement of the nuanced Afrocentric knowledge needed by students to best provide hearing care services in a contextually relevant, Afrocentric manner (Sonkqayi, 2024).

Curricular knowledge was perceived as overly structured, presenting an idealistic perspective of the profession, which emanated from poor recontextualisation of professional practice into educational content (Bernstein, 2000; Hordern, 2021). Teaching methods often ignored students’ lived realities, undermining their ability and approaches to meaningfully integrate and apply knowledge. As a result, BAFLS students felt that they were trained to serve middle-class, English-speaking patients, rather than the majority BAFLS population, a concern raised in previous work on epistemic transformation in health sciences education (Khoza-Shangase & Mophosho, 2018; Pillay & Kathard, 2015).

#### Linguicism and cultural marginalisation

Language emerged as a key site of marginalisation that exacerbated the abovementioned misalignment between the students and the curriculum. As Brunner (2021) notes, colonial languages can serve as a means of advancing epistemic violence, as appears to be the case in the current study. Despite national policies promoting multilingualism in higher education (DHET, 2017), isiZulu and other African languages remained peripheral in teaching and assessment. Language plays a dominant role in how students express themselves to clinical supervisors, lecturers and patients while engaged in activities that are assessed for marks, to their detriment if they best express themselves in African languages, while privileging those who are first-language English speakers. This linguicism, manifesting in the privileging of English, undermined students’ ability to demonstrate clinical competence during assessments and contributed to lower academic outcomes (Flores & Rosa, 2015; Mphasha et al., 2022).

Furthermore, the curriculum lacked cultural sensitivity outside Eurocentrism. Afrocentric worldviews, including beliefs around illness, healing and patient interaction norms, were notably absent. This omission reflects what Le Grange (2016) describes as the ‘null curriculum’, knowledge that is systematically excluded because of ideological or epistemological bias. This suggests that racial transformation in audiology has not yielded the necessary epistemic shift, rendering BAFLS students epistemic foreigners subjected to unjust assessment practices.

#### Assessment bias and epistemic injustice

Participants reported biased assessment practices that disproportionately affected BAFLS students. These included racially and linguistically biased grading, inconsistent clinical supervision and unequal expectations regarding clinical resources. Such experiences align with the concept of *epistemic injustice*, the denial of credibility based on identity-related prejudices (Bygren, 2020; Fricker, 2007).

Assessment practices appeared to reinforce institutional hierarchies by favouring English-speaking, middle-class students and penalising those from disadvantaged backgrounds. This not only affected students’ marks but also shaped their identities as learners and future professionals. The power asymmetries between students and supervisors further exacerbated these effects, as noted in both South African and international literature (Botelho et al., 2015; Jawitz, 2012). Hence, maladaptive coping mechanisms resulted.

### Surface learning as a coping mechanism

Confronted with structural barriers and discriminatory practices, many students adopted surface learning strategies. They described learning ‘to pass’ rather than to develop integrated clinical competence. This finding supports the argument that misaligned assessment practices, particularly those disconnected from students’ linguistic and cultural backgrounds, push students towards instrumental learning and impede critical engagement (Shah et al., 2016; Steen-Utheim & Hopfenbeck, 2019).

Surface learning was exacerbated by the volume of content and the lack of pedagogical strategies that supported integration and application. As a result, students felt ill-equipped to engage holistically with patients and often struggled to bridge the gap between theoretical and practical knowledge after graduation.

Overall, the above findings suggested not only a need for an epistemic shift but also a clearly defined criterion that will be used to benchmark the fundamental change. Fortunately, structures that can enforce compliance and manage non-compliance already exist. Yet, it seems they operate in silos and lack uniformity in terms of what constitutes a fundamental transformation. Hence, there is a need for each stakeholder contributing to the curriculum to identify their isolated roles and responsibilities in the implementation transformation and to account to the team that represents all other stakeholders. Those specific stakeholders include the institutional and national quality assurance bodies (Higher Education Council, Quality Promotion and Assurance), professional council (HPCSA), professional associations (National Black Speech-Language-Hearing Association, South African Speech-Language-Hearing Association, South African Association of Audiology), Audiologists and students.

### Transformative pathways: Towards an epistemically just curriculum

The findings underscore the urgent need for epistemic transformation within the Audiology curriculum. Specifically, there is a need to address three key areas.

To avoid the experiences of linguicism and ensure linguistic justice, institutions must move beyond tokenistic multilingualism and meaningfully integrate African languages into their teaching, learning and assessment practices. This includes valuing linguistic diversity as an asset rather than a deficit and resisting the hegemony of English as the sole language of epistemic legitimacy (Oliver & Exell, 2020; Oliver & Oliver, 2017). Practically, students should be able to choose to be assessed in isiZulu or English without related negative consequences.

To ensure Afrocentric curriculum recontextualisation, African knowledge systems must be foregrounded in curriculum design. This includes integrating cultural beliefs, community practices and local health epistemologies into Audiology training. Stakeholder engagement should chart a path towards exploring, recognising and legitimising African knowledge systems and extracting aspects that can benefit student learning, including Afrocentric pedagogies. Institutional quality assurance structures, health councils, associations, curriculum designers, the community and students are among the important stakeholders to be considered in the recontextualisation and knowledge legitimation processes. Recontextualising professional knowledge through an Afrocentric lens will ensure graduates are prepared for real-world service in public health sectors and diverse communities (Matiso, 2024; Pillay et al., 2024).

To enforce inclusive pedagogy and assessment reform, assessment practices must become fundamentally culturally responsive, transparent and student centred. Clinical supervisors and academic staff should be trained in anti-racist, anti-classist and anti-linguistic assessment practices. Such practices should not only be reflected in the planned but also in the experienced curriculum. As argued by Godsell et al. (2024), assessment is not merely a measurement tool but a site of pedagogic power; it can either reinforce colonial hierarchies or challenge them.

This study highlights how epistemic exclusion, linguistic injustice and structurally biased assessment practices compromise the learning and professional development of BAFLS students in Audiology. To address these challenges, higher education institutions must commit to a fundamental curriculum transformation that affirms the decolonial cultural, linguistic and socioeconomic identities of African students. Such change requires the collaboration of universities, professional councils and practising clinicians to radically reimagine how knowledge is constructed, validated and taught within the health sciences. Only then can undergraduate curricula produce Audiology graduates who are not only technically competent but also culturally attuned and socially responsive practitioners.

### Implications and recommendations

The findings of this study have several important implications for curriculum development, pedagogical reform and policy transformation within South African Audiology education, at universities where the study findings are applicable.

### Curriculum design and epistemic transformation

There is an urgent need for higher education institutions to engage in critical reflection on the design, structure and knowledge foundations of the undergraduate Audiology curriculum. This includes a deliberate and systematic process of epistemological transformation, one that moves beyond surface-level curriculum adjustments and towards genuine decolonisation and Africanisation. The curriculum must be reimagined to reflect local knowledge systems, patient realities and the sociocultural diversity of South Africa.

### Collaborative recontextualisation of professional knowledge

The process of recontextualising professional knowledge into undergraduate curricula should involve meaningful collaboration among key stakeholders. These include academic staff, professional and qualifications regulatory bodies, practising Audiologists, students and community representatives. This collaboration must ensure that curriculum content and pedagogical practices are responsive to the needs of diverse student populations and the public health realities they will serve.

### Policy development and accountability structures

A coherent and binding national policy framework is necessary to guide the transformation of health sciences curricula, with a specific emphasis on decolonial and inclusive pedagogies. This policy should outline standards, implementation strategies and clear transformation benchmarks. Furthermore, the establishment of independent monitoring and evaluation bodies, comprising representatives from academia, professional councils and civil society, is essential to ensure that institutional transformation is measurable, sustainable and transparent. A lack of compliance, measured against explicit criteria, should attract closer support and enforcement for those who lack the capacity to effectively implement the required changes. However, if no fundamental transformation is evident, even after the provided support, institutions and audiology disciplines in question should face a risk of losing accreditation.

### From rhetoric to practice

Ultimately, there is a pressing need to move beyond theoretical discussions of transformation and towards action-oriented reform. Institutions must avoid perpetuating a status quo that maintains epistemic and structural inequalities. Instead, tangible strategies, adequate resourcing and committed leadership are required to drive meaningful and lasting change in the higher education and health sciences sectors.

## Conclusion

This study has highlighted significant epistemic and pedagogical shortcomings within the undergraduate Audiology curriculum at the UoI, as experienced by BAFLS. The participants reported that the curriculum inadequately reflected the realities of clinical practice in South Africa and marginalised Afrocentric knowledge systems. Their experiences of biased assessment and culturally unresponsive teaching practices were interpreted as manifestations of racism, linguicism and classism. These structural inequities contributed to a sense of underpreparedness for delivering contextually relevant, decolonial and patient-centred audiology services upon graduation.

The findings underscore the urgent need for epistemological transformation that prioritises African knowledge systems, promotes inclusivity in curriculum design and assessment and addresses the systemic biases that continue to disadvantage historically marginalised students. While some progress towards racial representation may have occurred in the field of Audiology, deeper epistemic and curricular reform remains critical. The proposed pathways for transformation offer a concrete starting point for advancing equity and relevance in health professions education in South Africa.
